# A Comparative Study of Combined Spinal Epidural Anesthesia Versus Spinal Anesthesia in Major Lower Limb Orthopedic Surgeries

**DOI:** 10.7759/cureus.67354

**Published:** 2024-08-20

**Authors:** Ishan R Gadekar, Hukam S Rawat, Amreesh Paul

**Affiliations:** 1 Anaesthesiology, Jawaharlal Nehru Medical College, Datta Meghe Institute of Higher Education and Research, Wardha, IND; 2 Anaesthesiology, Dr. Vithalrao Vikhe Patil Institute of Medical Sciences and Research, Ahmednagar, IND

**Keywords:** combined spinal-epidural anesthesia, anesthetic complications, orthopedic surgery, anesthetic efficacy, postoperative outcomes, pain management, anesthesia techniques, major lower limb orthopedic surgeries, spinal anesthesia

## Abstract

Background

Neuraxial blockade includes epidural and spinal anesthesia (SA) that have gained wide acceptance for major lower limb orthopedic surgery. Both techniques are competent in rendering surgical anesthesia and pain relief, with specific advantages and disadvantages. SA has the merits of rapid onset and adequate anesthesia with a small volume of the drug but has significant hypotension and unpredictable duration. Epidural anesthesia allows for finer control over analgesia and the duration of anesthesia but requires more substantial volumes of drugs and is slower in onset. Combined spinal-epidural anesthesia (CSEA) combines the rapid commencement of action of SA with flexibility in epidural anesthesia, thus optimizing the management of the intraoperative and postoperative phases. This study aims to evaluate hemodynamic changes, compare the severity and duration of sensory and motor block, and track any problems related to CSEA and SA in major lower limb orthopedic procedures. Additionally, this study contrasts the hemodynamic, motor, and sensory changes in the two groups.

Methodology

A total of 30 individuals were randomized to one of two groups in this prospective comparative trial, which included 60 patients receiving major lower limb orthopedic surgery and meeting the American Society of Anesthesiologists physical status I-II criteria. Group A received CSEA, and Group B received SA. The degree and duration of sensory and motor blockade, hemodynamic changes, and complications were all recorded. A p-value of less than 0.05 was used to evaluate statistical significance using Student’s t-test and chi-square test.

Results

The onset of sensory block in our study was earlier in Group B compared to Group A. In both groups, hemodynamic stability was maintained throughout the study. We recorded the onset/duration of sensory and motor block and hemodynamic changes and took mean values to find any significant difference. Postoperative complications and rescue analgesic requirements were monitored and managed and were a part of our study.

Conclusions

This study compared CSEA and SA regarding the severity and duration of sensory and motor block, hemodynamic stability, and associated complications in major lower limb orthopedic surgeries. The results shed light on the advantages and shortcomings of each anesthesia technique and, therefore, will help choose the correct method of anesthesia in a given surgery.

## Introduction

Over the past few decades, the neuraxial blockade has been employed extensively for major lower limb orthopedic procedures. Spinal and epidural blockade have long been shown to be beneficial for a variety of surgical procedures as well as pain management. They can result in sensorimotor, sympathetic, motor blockade, or any combination of these effects depending on the volume, concentration, and amount of local anesthetic employed. Furthermore, central neuraxial blocking lowers the risk of thromboembolic events, intraoperative blood loss, the stress response to surgery, and death and morbidity in high-risk surgical patients [1].

August Bier used the local anesthetic cocaine to accomplish the first human spinal anesthesia (SA) in 1898 [2]. Other local anesthetics have since been utilized. Emblem used bupivacaine for the first time in 1966 [3]. SA is a low-cost, dependable, and safe method. Its quick onset of action and efficient surgical anesthesia is achieved with a minimal volume of medication. However, it comes with the disadvantages of severe hypotension and the inability to plan and regulate the length of anesthesia [4].

Because epidural anesthesia allows for more control over the length of anesthesia as well as better control over analgesia during and after surgery, it is preferable. Contrarily, epidural anesthesia necessitates a significant amount of local anesthetic to create pharmacologically active systemic blood levels. This may result in difficulties and side effects that are not seen with SA. However, it has drawbacks, such as a somewhat delayed commencement of action and sporadic patchy anesthesia [5]. Combining spinal and epidural procedures has blurred certain distinctions while increasing clinical care flexibility. Although combined spinal-epidural anesthesia (CSEA) was initially published in 1937, it has since undergone modifications and is currently gaining favor. The operative procedure can start earlier because spinal block occurs more quickly in CSEA than in epidural anesthesia [6]. In contrast, an epidural catheter still offers adequate postoperative analgesia and permits the extension of anesthesia as the effects of SA wears off. In the sequential combination spinal epidural approach, a smaller dose of local anesthetic is first injected into the subarachnoid space. If a more epidural local anesthetic is subsequently required to reach a higher degree of the block, it is then injected [7]. It combines the flexibility of an epidural catheter with the quick onset and potent analgesia of a subarachnoid block, all the while utilizing a much smaller amount of anesthetic than is required for epidural anesthesia at the same level, which minimizes adverse effects. In addition to allowing for quicker recovery and increased hemodynamic stability for high-risk patients, this sequential method may shorten hospital stays [8].

Measuring the degree and duration of the sensory and motor block using CSEA is the main objective of this study. Secondary goals include comparing the two groups’ changes in sensory, motor, and hemodynamic parameters; measuring the intensity and duration of the sensory and motor block; observing hemodynamic variations associated with both SA and CSEA; and monitoring any adverse effects with the two techniques, as previously mentioned.

## Materials and methods

During the course of 18 months following the Institutional Ethics Committee’s clearance date, a prospective comparative trial was carried out. A total of 60 patients with the American Society of Anesthesiologists (ASA) physical status I-II who were scheduled for major lower limb orthopedic surgery were included after obtaining informed consent in writing. The study’s inclusion criteria included patients of any sex who were scheduled for elective major lower limb orthopedic surgery, had an ASA physical status of I-II, weighed between 50 and 70 kg, were willing to participate, and gave written informed consent. The patient’s refusal of the surgery, allergies to amide anesthetics, any contraindications to the study methods, and extremes in height or weight were the exclusion criteria.

Sample size calculation

A minimum sample size of 44, with 22 patients in each group was calculated using Openepi.com, Version 3, an open-source calculator with a confidence interval of 95%, a power of 80%, and a sample size ratio of 1 between both groups. The sample size was calculated using the mean and standard deviation of the total duration of analgesia given by CSEA and SA in a study by Shah et al. [9], as shown in Table 1. To account for possible dropouts during the study, 60 patients were recruited.

**Table 1:** Sample size calculation. Sample size calculated using OpenEpi.com.

	Group 1	Group 2	Difference between means
Mean	3.25	5.07	-1.82
Standard deviation	0.41	0.55	
Variance	0.1681	0.3025	

Randomization and blinding

Randomization was achieved using a set of sealed opaque envelopes containing allo­cation cards prepared by the statistician not involved in patient care. Each envelope was sequentially numbered and inside had a card stating either Group A or Group B. Eligible patients were given the next sequentially numbered envelope indicating their group assignment as soon as they arrived in the preoperative area. Patients in Group B were given SA, whereas patients in Group A were given CSEA. Because of the nature of the interventions, blinding of patients and anesthesia providers was impossible. The outcome assessors and data analysts were blinded throughout the study period to reduce bias.

Methodology

The patients were on an overnight fast as part of the anesthesia technique. The day before the procedure, they underwent a pre-anesthesia check-up, during which routine and specialized investigations were conducted. The patients’ issues were addressed, and the study’s methodology was explained. Patients received an intravenous injection of ondansetron 4 mg 30 minutes before surgery, but no sedative or opioid premedication was administered before they arrived in the operating room. Upon arrival at the operation theater (OT), temperature, blood pressure, pulse rate, ECG, and SpO_2_ were measured for all patients. Ringer’s lactate solution was preloaded into the intravenous (IV) lines of the patients 20 minutes before surgery, with an 18 G Intracath used to secure the line.

The OT assistant assisted in placing the patients in Group A in the sitting posture. An OT fabric was draped after disinfecting the lumbar area with Savlon, betadine, and spirit. A cutaneous weal elevated on 2% lignocaine was detected in the L3-L4 region. An air-filled syringe and the loss of resistance approach were utilized to insert an 18 G Tuohy needle into the epidural area. Following the insertion of the needle, the epidural catheter was positioned 2-4 cm into the epidural space, fastened, and aspirated to examine for evidence of blood or cerebrospinal fluid. Using a 25 G Quincke spinal needle, 1.4 mL of 0.5% hyperbaric bupivacaine was injected to administer SA. The medication was delivered into the cerebrospinal fluid, and then the spinal needle was removed. Gradually, the patient was put in a supine position. To extend the block to T10, 1.0-1.5 mL of 0.5% isobaric bupivacaine was given by an epidural route for each segment that was unblocked. The pinprick test was used every five minutes to demonstrate the loss of sensation until the block’s maximum value was reached and then every 15 minutes.

In Group B, a 25 G Quincke spinal needle was utilized to inject 3 mL of 0.5% hyperbaric bupivacaine into the subarachnoid area while the patient was seated and completely aseptic. The medication was allowed to be fixed after the spinal needle was removed. Patients were gradually made supine for a progressive start to blocking. Sensory alterations were scored bilaterally after 10 minutes using a safety pin that protruded 2 mm through a guard. Normal feeling received a score of 0, muted sensation received a score of 1, and no sensation received a score of 2. This was followed by the start of the procedure, during which critical parameters were tracked. Following surgery, they were sent to the post-anesthesia care unit and then to the ward. When the Visual Analog Scale (VAS) score in Group A reached 4 during the postoperative phase, an epidural top-up comprising 8 cc of 0.125% levobupivacaine and 50 mg of tramadol was administered. Group B received intramuscular diclofenac as needed to relieve pain.

Study parameters

Examination was done on the length of time and the commencement of the motor and sensory block. Heart rate (HR), diastolic blood pressure (DBP), mean arterial pressure (MAP), systolic blood pressure (SBP), and SpO_2_ were assessed. Utilizing a modified Bromage scale, the motor block was assessed [10]. The level of sensory block was measured using VAS [11]. During the first two hours following surgery, all patients were observed every 15 minutes. Subsequently, they were observed every three hours, and, finally, every two hours for 12 hours. Twelve hours were spent noting all the factors and scores, and 24 hours were spent monitoring the complications. During the first 12 hours following surgery, rescue analgesic need was observed. IV fluids were administered as needed. Postoperative problems, including bradycardia, hypotension, respiratory depression, nausea, pruritus, vomiting, and arrhythmias, were closely monitored in all patients. In addition to noting the sensory and motor block length, postoperative problems were reported and appropriately addressed.

## Results

The demographic distribution of the two groups is displayed in Table 2. The mean age of Group A was 50.87 ± 6.942 years, whereas of Group B, it was 50.10 ± 6.445 years, with an insignificant p-value of 0.66. The mean weight of Group A was 58.30 ± 6.001 kg, whereas it was 57.93 ± 6.533 kg in Group B, with a p-value of 0.82 indicating no statistical significance. In Group A, the mean height was 159.23 ± 4.337 cm, with a p-value of 0.49, whereas in Group B, it was 158.33 ± 5.548 cm, which was also not statistically significant. The mean body mass index (BMI) was 22.966 ± 1.876 kg/m² in Group A, whereas it was 23.035 ± 1.422 kg/m² in Group B. The p-values were 0.87 for both groups, indicating they were not statistically significant.

**Table 2:** Demographic distribution. Group A = combined spinal-epidural anesthesia; Group B = spinal anesthesia. N = number of participants; BMI = body mass index

	Group	N	Mean	Standard deviation	P-value
Age	A	30	50.87	6.942	0.66
	B	30	50.10	6.445	
Weight	A	30	58.30	6.001	0.82
	B	30	57.93	6.533	
Height	A	30	159.23	4.337	0.49
	B	30	158.33	5.548	
BMI	A	30	22.966	1.876	0.87
	B	30	23.035	1.422	

The types of procedures performed are compiled in Table 3. Because of the similar distribution in both groups, there were no appreciable variations in the types of procedures performed.

**Table 3:** Type of surgery. Group A = combined spinal-epidural anesthesia; Group B = spinal anesthesia. CRIF = closed reduction and internal fixation; ORIF = open reduction and internal fixation; PFN = proximal femoral nailing

Type of surgery	Group		Total
	A	B	
Cemented left hip hemiarthroplasty	2	2	4
Cemented right hip hemiarthroplasty	1	2	3
CRIF left femur nailing	1	0	1
CRIF left tibia nailing	3	3	6
CRIF right femur nailing	2	3	5
CRIF right tibia nailing	5	3	8
CRIF with PFN left hip	1	2	3
CRIF with PFN right hip	4	2	6
ORIF left femur plating	4	5	9
ORIF left tibia plating	0	2	2
ORIF right femur plating	3	2	5
ORIF right tibia plating	4	4	8
Total	30	30	60

The average time of sensory block onset was 3.0 ± 0.947 minutes in Group A and 2.47 ± 0.681 minutes in Group B. The average time for the beginning of a motor block was 4.13 ± 0.86 minutes in Group A and 3.97 ± 0.809 minutes in Group B. Consequently, Group B had motor and sensory blockage before Group A. For sensory block, the difference was statistically significant, but not for motor block. Group A’s sensory block lasted 218 ± 29.665 minutes, while Group B’s lasted 172.73 ± 13.188 minutes. In Group A, the motor block lasted 195.67 ± 30.913 minutes, while in Group B, it lasted 150.7 ± 14.568 minutes. Table 4 demonstrates that Group A had sensory and motor blockades for significantly longer durations than Group B, with a statistically significant difference.

**Table 4:** Onset and duration of sensory and motor block. Group A = combined spinal-epidural anesthesia; Group B = spinal anesthesia. N = number of participants

	Group	N	Mean	Standard deviation	P-value
Onset of sensory block	A	30	3.00	0.947	0.015
	B	30	2.47	0.681	
Onset of motor block	A	30	4.13	0.860	0.443
	B	30	3.97	0.809	
Duration of sensory block (minutes)	A	30	218.00	29.665	<0.001
	B	30	172.73	13.188	
Duration of motor block (minutes)	A	30	195.67	30.913	<0.001
	B	30	150.70	14.568	

The p-values for HR, SBP, DBP, and MAP during all time points remained above the significance level, indicating that the difference in means observed in both groups was statistically insignificant. Both groups showed very similar physiology concerning HR and blood pressure measures, proving neither the conditions nor the interventions in either group resulted in vastly differing outcomes for the above measures. The comparison between HR, SBP, DBP, and MAP is presented in Tables 5-8, respectively. All patients in our study were hemodynamically stable preoperatively.

**Table 5:** Heart rate of both groups. Group A = combined spinal-epidural anesthesia; Group B = spinal anesthesia. N = number of participants; HR = heart rate

	Group	N	Mean	Standard deviation	P-value
HR 0 minutes	A	30	77.73	5.795	0.841
	B	30	78.07	6.977	
HR 15 minutes	A	30	73.57	5.600	0.796
	B	30	73.13	7.195	
HR 30 minutes	A	30	74.67	5.261	0.684
	B	30	74.80	5.092	
HR 45 minutes	A	30	75.33	5.416	0.532
	B	30	74.53	5.021	
HR 60 minutes	A	30	76.47	5.649	0.215
	B	30	74.47	6.658	
HR 75 minutes	A	30	77.27	5.836	0.899
	B	30	77.47	6.323	
HR 90 minutes	A	30	77.80	5.294	0.660
	B	30	78.47	6.339	
HR 105 minutes	A	30	78.67	5.665	0.869
	B	30	78.40	6.775	
HR 120 minutes	A	30	77.93	5.265	0.728
	B	30	78.47	6.511	
HR 180 minutes	A	30	78.80	5.215	0.281
	B	30	80.47	6.574	
HR 240minutes	A	30	80.20	5.442	0.935
	B	29	80.07	6.729	
HR 300 minutes	A	30	79.80	5.857	0.318
	B	30	78.20	6.440	
HR 420 minutes	A	30	79.10	5.756	0.520
	B	30	78.07	6.570	
HR 540 minutes	A	30	79.13	5.649	0.762
	B	30	78.67	6.200	
HR 660 minutes	A	30	80.67	4.766	0.251
	B	30	79.00	6.275	
HR 720 minutes	A	30	81.80	4.708	0.277
	B	30	80.20	6.462	

**Table 6:** Systolic blood pressure of both groups. Group A = combined spinal-epidural anesthesia; Group B = spinal anesthesia. N = number of participants; SBP = systolic blood pressure

	Group	N	Mean	Standard deviation	P-value
SBP 0 minutes	A	30	121.60	6.610	0.759
	B	30	122.13	6.766	
SBP 15 minutes	A	30	114.07	5.247	0.120
	B	30	110.33	5.851	
SBP 30 minutes	A	30	115.13	5.935	0.121
	B	30	118.60	6.116	
SBP 45 minutes	A	30	117.93	5.930	0.431
	B	30	116.20	5.160	
SBP 60 minutes	A	30	119.33	7.341	0.493
	B	30	117.93	8.346	
SBP 75 minutes	A	30	121.40	6.038	0.535
	B	30	120.87	7.157	
SBP 90 minutes	A	30	122.00	5.754	0.581
	B	30	122.87	6.318	
SBP 105 minutes	A	30	122.67	5.390	0.963
	B	30	122.60	5.805	
SBP 120 minutes	A	30	122.60	5.177	0.547
	B	30	123.47	5.871	
SBP 180 minutes	A	30	124.27	4.920	0.192
	B	30	126.00	5.252	
SBP 240 minutes	A	30	125.80	4.278	0.263
	B	30	124.40	5.263	
SBP 300 minutes	A	30	125.40	5.946	0.288
	B	30	123.93	4.563	
SBP 420 minutes	A	30	123.73	6.028	0.782
	B	30	124.13	5.063	
SBP 540 minutes	A	30	123.73	6.097	0.855
	B	30	124.00	5.092	
SBP 660 minutes	A	30	124.07	5.595	0.283
	B	30	125.53	4.862	
SBP 720 minutes	A	30	126.67	4.559	0.900
	B	30	126.80	3.547	

**Table 7:** Diastolic blood pressure of both groups. Group A = combined spinal-epidural anesthesia; Group B = spinal anesthesia. N = number of participants; SBP = diastolic blood pressure

	Group	N	Mean	Standard deviation	P-value
DBP 0 minutes	A	30	75.87	3.711	0.300
	B	30	77.13	5.501	
DBP 15 minutes	A	30	72.80	3.537	0.234
	B	30	71.33	6.019	
DBP 30 minutes	A	30	72.40	4.280	0.461
	B	30	71.80	5.517	
DBP 45 minutes	A	30	75.00	4.194	0.244
	B	30	74.33	5.261	
DBP 60 minutes	A	30	76.00	3.895	0.205
	B	30	74.47	5.270	
DBP 75 minutes	A	30	76.53	3.192	0.847
	B	30	76.73	4.683	
DBP 90 minutes	A	30	76.33	3.898	0.530
	B	30	77.00	4.259	
DBP 105 minutes	A	30	77.47	3.998	0.374
	B	30	76.33	5.659	
DBP 120 minutes	A	30	76.93	4.417	0.739
	B	30	76.53	4.840	
DBP 180 minutes	A	30	78.07	3.581	0.794
	B	30	77.80	4.278	
DBP 240 minutes	A	30	77.87	3.785	0.493
	B	30	77.13	4.416	
DBP 300 minutes	A	30	78.20	3.836	0.175
	B	30	76.73	4.409	
DBP 420 minutes	A	30	78.00	3.523	0.202
	B	30	76.67	4.436	
DBP 540 minutes	A	30	78.33	3.898	0.142
	B	30	76.60	5.042	
DBP 660 minutes	A	30	78.73	3.877	0.084
	B	30	76.73	4.884	
DBP 720 minutes	A	30	78.87	3.702	0.241
	B	30	77.67	4.138	

**Table 8:** Mean arterial pressure of both groups. Group A = combined spinal-epidural anesthesia; Group B = spinal anesthesia. N = number of participants; MAP = mean arterial pressure

	Group	N	Mean	Standard deviation	P-value
MAP 0 minutes	A	30	91.11	4.13	0.348
	B	30	92.13	4.24	
MAP 15 minutes	A	30	84.56	3.79	0.838
	B	30	84.33	4.54	
MAP 30 minutes	A	30	86.64	4.02	0.241
	B	30	84.73	4.98	
MAP 45 minutes	A	30	89.31	4.13	0.142
	B	30	87.29	4.95	
MAP 60 minutes	A	30	90.44	4.39	0.222
	B	30	88.96	4.94	
MAP 75 minutes	A	30	91.49	3.57	0.630
	B	30	91.98	4.23	
MAP 90 minutes	A	30	91.56	3.88	0.432
	B	30	92.29	3.27	
MAP 105 minutes	A	30	92.53	3.73	0.454
	B	30	91.76	4.24	
MAP 120 minutes	A	30	92.16	3.91	0.982
	B	30	92.18	3.84	
MAP 180 minutes	A	30	93.47	3.39	0.663
	B	30	93.87	3.68	
MAP 240 minutes	A	30	93.84	3.50	0.297
	B	30	92.89	3.53	
MAP 300 minutes	A	30	93.93	3.81	0.106
	B	30	92.47	3.06	
MAP 420 minutes	A	30	93.24	3.49	0.415
	B	30	92.49	3.63	
MAP 540 minutes	A	30	93.47	3.78	0.291
	B	30	92.40	3.97	
MAP 660 minutes	A	30	93.84	3.49	0.372
	B	30	93.00	3.77	
MAP 720 minutes	A	30	94.80	3.16	0.354
	B	30	94.04	3.11	

Table 9 reveals that at 0 minutes, groups A and B had mean pain scores of 5.47 and 5.50, respectively, with a p-value of 0.8. At 15, 30, 45, 60, 75, 90, 105, and 120 minutes, neither group had any pain, as shown by a VAS of 0 with 0 standard deviations, making any statistical comparison meaningless. With a p-value <0.001, Group A’s mean VAS score at 180 minutes was 0.13, significantly lower than Group B’s mean VAS score of 2.47. The observed mean VAS in Group A was 0.40 at 420 minutes, with a p-value <0.001; at 540 minutes, 0.00 versus 1.67, with a p-value <0.001; at 660 minutes, 0.33 versus 1.40, with a p-value <0.005; and at 720 minutes, 0.63 versus 1.23, with a p-value <0.001. These results demonstrate that Group A had significantly less pain than Group B did at different times after the surgery.

**Table 9:** VAS scores at various time intervals. Group A = combined spinal-epidural anesthesia; Group B = spinal anesthesia. ^a^: As the standard deviations of both groups are zero, t cannot be calculated. N = number of participants; VAS = Visual Analog Scale

	Group	N	Mean	Standard deviation	P-value
VAS 0 minutes	A	30	5.47	0.507	0.8
	B	30	5.50	0.509	
VAS 15 minutes	A	30	0.00	0.000^a^	-
	B	30	0.00	0.000^a^	
VAS 30 minutes	A	30	0.00	0.000^a^	-
	B	30	0.00	0.000^a^	
VAS 45 minutes	A	30	0.00	0.000^a^	-
	B	30	0.00	0.000^a^	
VAS 60 minutes	A	30	0.00	0.000^a^	-
	B	30	0.00	0.000^a^	
VAS 75 minutes	A	30	0.00	0.000^a^	-
	B	30	0.00	0.000^a^	
VAS 90 minutes	A	30	0.00	0.000^a^	-
	B	30	0.00	0.000^a^	
VAS 105 minutes	A	30	0.00	0.000^a^	-
	B	30	0.00	0.000^a^	
VAS 120 minutes	A	30	0.00	0.000^a^	-
	B	30	0.00	0.000^a^	
VAS 180 minutes	A	30	0.13	0.730	<0.001
	B	30	2.47	1.737	
VAS 240 minutes	A	30	2.33	1.668	0.005
	B	30	3.30	0.750	
VAS 300 minutes	A	30	1.93	0.929	0.005
	B	30	2.50	0.572	
VAS 420 minutes	A	30	0.40	1.221	<0.001
	B	30	2.07	0.691	
VAS 540 minutes	A	30	0.00	0.000	<0.001
	B	30	1.67	0.547	
VAS 660 minutes	A	30	0.33	0.711	<0.001
	B	30	1.40	0.498	
VAS 720 minutes	A	30	0.63	0.765	<0.001
	B	30	1.23	0.430	

Regarding postoperative complications, three patients in Group B reported nausea, while no patients in Group A reported the same. Gour patients in Group B had headaches compared to one patient in Group A. Both anesthetic procedures were safe because neither group experienced life-threatening intraoperative or postoperative adverse effects. Significant systemic adverse effects following surgery were less common in Group A (CSEA). Group A consisted of only one patient who experienced partial motor paresis, which resolved on its own without causing any systemic side effects. Therefore, CSEA offers more advantages than disadvantages.

## Discussion

Our study examined CSEA and SA in patients requiring major orthopedic procedures on their lower limbs. Regarding demographic information and hemodynamic characteristics, the patients in Group A and Group were similar. These findings are comparable to those of previous studies [8,12-15]. Group A showed gradual reductions in SBP, DBP, and MAP parameters. Thus, a sudden fall in blood pressure after induction did not happen in Group A. Greater hemodynamic stability was observed throughout the intraoperative period in Group A patients. Bradycardia was not observed. All patients in Group A achieved baseline hemodynamics well before the end of the intraoperative period. Group B showed significant reductions in HR, SBP, DBP, and MAP parameters. A sudden fall in blood pressure was observed in Group B over time, which took considerable time to recover to baseline hemodynamics. Some patients had to be supplemented with vasopressors during 15 minutes post-induction. Some patients also showed bradycardia, which was treated with vagolytics. Some patients did not achieve baseline hemodynamics at the end of the intraoperative period. As a result, during the intraoperative phase, Group A’s hemodynamics were more stable due to CSEA than Group B’s due to SA. Bradycardia, which was noted in Group B, was likewise not present in Group A. In addition, Group A recovered to baseline hemodynamics earlier than Group B. This finding aligned with previous studies [8,12-14].

In terms of statistical significance, the mean time of the sensory block beginning was observed. The onset of sensory block for hyperbaric bupivacaine was reported to be two to three minutes in the study by Whiteside et al. on hyperbaric bupivacaine and hyperbaric ropivacaine, which is analogous to our research [16]. The commencement of sensory block for hyperbaric bupivacaine was reported to be 76.67 ± 16.51 seconds, which is about 1.3 minutes, by Sapate et al. on subarachnoid hyperbaric bupivacaine-clonidine and hyperbaric bupivacaine, which coincides with our study [17]. In a study by Mohamed et al. on dexmedetomidine as an adjuvant to lower doses of intrathecal bupivacaine for lower limb orthopedic surgeries, the onset of sensory blockade was observed in all groups within two to three minutes (Group A: 2.33 ± 0.568 minutes, Group B: 2.10 ± 0.305 minutes, Group C: 2.00 ± 0.025 minutes), which is consistent with our findings [18]. In SA, conventional hyperbaric bupivacaine and low-dose bupivacaine-fentanyl for orthopedic surgeries were evaluated in a study by Mehta et al. [19]. Consistent with our findings, the study demonstrated that the typical bupivacaine group experienced an approximately 1.6-minute onset of sensory blockage. In the study by Bhattacharya et al., which compared sequential CSEA with SA in high-risk elderly patients undergoing major lower limb orthopedic surgery, the start of sensory block was 10.10 ± 1.1 minutes for Group A and 9.8 ± 1 minutes for Group B. These findings did not align with the findings of our investigation [20]. Our study offers an advantage with a significantly shorter time of onset by using low-dose hyperbaric bupivacaine intrathecally. In a study by Koch et al. evaluating levobupivacaine for epidural anesthesia and postoperative analgesia in hip surgeries, the time to sensory block with levobupivacaine was 18.6 ± 15.9 minutes, and with bupivacaine was 17.9 ± 14.2 minutes [21]. The average duration from the first anesthetic injection to the point at which major orthopedic surgery could be performed, as determined by Casati et al., was 27 minutes [22]. Because CSEA was used in our investigation, there was an advantage in terms of an earlier onset of sensory and motor blockade and a shorter recovery period following surgery. In both groups, the start of sensory blockade did not differ significantly. Patients in Group B attained sensory blockade sooner. However, the preparedness of both groups for surgery time was unaffected by this. Group A successfully initiated a sensory blockade at a suitable point.

The length of sensory blockade was statistically very significant in our investigation (p < 0.001). In accordance with our findings, Bhattacharya et al. compared sequential CSEA versus SA in high-risk elderly patients undergoing major lower limb orthopedic surgery and found that the CSEA group’s mean analgesic duration was 260 ± 10 minutes, whereas Group B’s was 190 ± 10 minutes [20]. Consistent with our investigation, the study by Vives et al. on elderly patients receiving SA during orthopedic surgery for hip fractures indicated that the bupivacaine group’s sensory blockade lasted 178.25 ± 44.89 minutes. The duration of analgesia for patients undergoing lower abdominal urological and lower limb orthopedic surgeries was 92.32 ± 8.34 minutes in a study by Haque et al. comparing the efficacy of bupivacaine and bupivacaine-dexamethasone in SA [23]. This finding is inconsistent with the mean duration of analgesia noted in our study. The mean duration of sensory blockade for a conventional dose of bupivacaine group was reported to be 227.6 ± 9.8 minutes in a study by Mehta et al. comparing a low dose of bupivacaine-fentanyl versus a conventional dose of hyperbaric bupivacaine in SA for orthopedic surgery [19]. Despite using the same amount of medicine at the same concentration, Group B in our study had a duration of 172.73 ± 13.188 minutes. The length of analgesia for the bupivacaine control group was 204.80 ± 16.81 minutes, which is consistent with the findings of Group B in our study. The effects of intrathecal clonidine and dexmedetomidine on the properties of bupivacaine spinal block for lower limb procedures were compared in a study by Sarma et al. [24]. A significant variation was seen in the duration of the sensory block between Group A and Group B. Group A offered sensory blockade for a noticeably longer period, lasting well into the recovery phase. Consequently, Group A attained VAS values later than Group B. In Group A, this resulted in a longer time to rescue analgesia.

The start of the motor blockade was not statistically significant in our investigation (p = 0.443). The amount of medication given in Group B may have contributed to the earlier time to Bromage 1. The length of motor obstruction was statistically significant (p < 0.001) in our investigation. Similar to our study, the study by Vives et al. on elderly patients receiving SA after orthopedic surgery for hip fractures found that Group B’s motor blockade lasted 157.04 ± 41.71 minutes [25]. The mean duration of motor blockade for a conventional dose of bupivacaine group during orthopedic surgery in SA was 162.5 ± 7.5 minutes in the study by Mehta et al. comparing a low dose of bupivacaine-fentanyl to a conventional dose of hyperbaric bupivacaine [19]. This motor blockade duration matched our findings. Both groups saw a comparable onset of motor blockade, which was not statistically significant. There was a substantial difference between the motor blockade duration of Group A and Group B. Patients in Group B experienced motor recovery sooner. Though both groups acquired motor capabilities well before the time to ambulate following surgery, this quicker recovery did not provide any advantage in terms of time to ambulation. These findings are consistent with previous studies [13,15,15,20]. It took Group A much longer to reach a VAS score of 4. Clinically and statistically meaningful pain relief was maintained by Group A patients for a longer period following surgery. Therefore, rescue analgesia was needed for these individuals far later than for Group B. In the postoperative phase, Group A also displayed a superior analgesic profile compared to Group B. Lower VAS scores than Group B up to 12 hours after induction demonstrated this. As a result, in terms of analgesia, Group A patients experienced greater postoperative comfort than Group B patients. There was also the drawback of giving Group B patients an intramuscular injection as an additional invasive analgesia. These findings were in line with previous research [15,20,26].

Three patients in Group B complained of nausea throughout the postoperative period, whereas Group A patients did not report this symptom. None of the patients in either group reported any episodes of vomiting. Only one patient from Group A had headaches following surgery. Four patients in Group B also reported this postoperative complaint, which could be linked to the side effects of diclofenac. As a result, the general safety of SA and CSEA was demonstrated, as neither group experienced any potentially fatal side effects during the intraoperative or postoperative phases. On the other hand, patients in Group A experienced systemic side effects at a considerably lower level than those in Group B. Only one patient in Group A who had an epidural top-up showed signs of partial motor paresis of Bromage 1 grade. This patient recovered on its own in a few hours without any further issues. Therefore, CSEA may have a better safety profile than SA given the decrease in systemic adverse effects in Group A. These results are similar to those reported in previous studies [9,12,13,15,26].

This study has various limitations, which, while interpreting the results of this study, need to be kept in mind. First, due to the nature of interventions, i.e., CSEA versus SA, it was not possible to blind the patient or the anesthesia provider. Hence, bias may occur in subjective evaluations such as pain scores. Second, although an attempt at standardization of anesthesia techniques was made, variations in procedural minutiae could impact the uniformity of treatments. Although the sample size was calculated as per the minimum requirements, it remains relatively small and may not capture the entirety of the patient demographic or surgical complexity. These limitations raise areas for consideration in future studies aiming to compare anesthesia methods for major lower limb orthopedic surgeries.

## Conclusions

In this study, we compared CSEA versus SA for major lower limb orthopedic surgeries. Patient characteristics were comparable in both groups concerning age, height, weight, and BMI. Analgesia had a slightly faster onset in the SA group but was considerably longer in the CSEA group. The SA group experienced analgesia significantly sooner, but the CSEA group sustained it for far longer. While both groups experienced a similar motor blockade onset, the CSEA group’s block duration was noticeably greater. The hemodynamic parameters in the CSEA group exhibited a progressive decline in SBP, DBP, and MAP, whereas the abrupt decline in the SA group was accompanied by bradycardia. Analgesia during the postoperative period and patient comfort by CSEA were far superior to those in the SA group, with fewer episodes of nausea. While both groups presented headaches, there were more undesirable effects in the SA group. In general, these findings indicate that while both CSEA and SA are safe and efficient methods of anesthesia, the former may have fewer systemic side effects during the postoperative phase of managing patients, such as aiding in comfort and recovery. The benefits of CSEA, as indicated by a lower incidence of nausea, headaches, and systemic side effects, seem to outweigh the minor and transient disadvantage of partial motor paresis that was noted in only one patient.
